# Faeco-prevalence of *Campylobacter jejuni* in urban wild birds and pets in New Zealand

**DOI:** 10.1186/1756-0500-8-1

**Published:** 2015-02-02

**Authors:** Vathsala Mohan

**Affiliations:** Institute of Veterinary, Animal and Biomedical Sciences, Massey University, Palmerston North, New Zealand; Post-Doctoral Scientist, AgResearch, Grasslands, Palmerston North, New Zealand

**Keywords:** *Campylobacter* spp, *C. jejuni*, Urban wild birds, Pets, Faeco-prevalence

## Abstract

**Background:**

Greater attention has been given to *Campylobacter jejuni* (*C. jejuni*) prevalence in poultry and ruminants as they are regarded as the major contributing reservoirs of human campylobacteriosis. However, relatively little work has been done to assess the prevalence in urban wild birds and pets in New Zealand, a country with the highest campylobacteriosis notification rates. Therefore, the aim of the study was to assess the faeco-prevalence of *C. jejuni* in urban wild birds and pets and its temporal trend in the Manawatu region of New Zealand.

**Findings:**

A repeated cross-sectional study was conducted from April 2008 to July 2009, where faecal samples were collected from 906 ducks, 835 starlings, 23 Canadian goose, 2 swans, 2 pied stilts, 498 dogs and 82 cats. The faeco-prevalence of *C. jejuni* was 20% in ducks, 18% in starlings, 9% in Canadian goose, 5% in dogs and 7% in cats. The faeco-prevalence of *C. jejuni* was relatively higher during warmer months of the year in ducks, starlings and dogs while starlings showed increased winter prevalence. No such trend could be assessed in Canadian goose, swans, pied stilts and cats as samples could not be collected for the entire study period from these species.

**Conclusions:**

This study estimated the faeco-prevalence of *C. jejuni* in different animal species where the prevalence was relatively high during warmer months in general. However, there was relative increase in winter prevalence in starlings. The urban wild bird species and pets may be considered potential risk factors for human campylobacteriosis in New Zealand, particularly in small children.

## Background

*Campylobacter* spp. is one of the major causes of bacterial gastroenteritis worldwide [1], where the majority of the human campylobacteriosis cases are attributed to *C. jejuni* with *C. coli* and *C. upsaliensis* being isolated in small proportion of human cases [2–5]. The disease is self-limiting, however, severe sequelae such as Guillain-Barre syndrome and reactive arthritis have been recorded occasionally [6]. The risk of human campylobacteriosis arising from food source has been extensively studied [7–11] where the risk of environmental exposure to faecal material from livestock, including wild birds, ruminants and pet animals is under studied [12, 13]. Different exposure pathways have to be elucidated to better understand the relative contributions of various environmental factors towards human campylobacteriosis. Previous studies on urban wild birds and pet animals have identified the link between the *C. jejuni* populations from wild birds and human campylobacteriosis [14]. Urban wild birds including ducks, goose, swans and starlings cause enormous faecal contamination of the environment where population biology studies of Campylobacter conducted in ducks, goose and starlings have shown that these bird species cannot be excluded from being contributing sources of Campylobacter infection for farm animals and humans [15, 16]. Therefore, the present study was aimed to estimate the faeco-prevalence of *C. jejuni* in ducks, goose, swans and starlings from urban wild bird species and from dogs and cats in the Manawatu region of lower north island of New Zealand.

## Findings

### Research aim

The research aim was to estimate the faeco-prevalence of *Campylobacter* and *C. jejuni* in urban wild birds and pets at different sampling sites and over different time periods.

## Methods

### Study design

A repeated cross sectional study was conducted for sixteen months from April 2008 to July 2009. Five public parkland sites within the city limits were selected for sampling duck, goose, swan and starling faecal material: The Square, Hokowhitu, Memorial Park, Massey University and The Esplanade; all sites had at least one duck pond. To calculate the sample size for the urban wild birds, the prevalence was assumed in the order of 30-35% based on the previous report [16]. Each of the five study sites was visited at monthly intervals for a period of 17 months, starting in March 2008, where an initial standardisation for sample collection, transport and culture was carried out during March 2008 sampling round. For dogs and cats 50% design prevalence was assumed for the sample size calculations due to the wide range of prevalence estimates in the previous studies [17–21]. A 95% level of confidence and a 5% margin of error [22] were used for all the sample size calculations. Ten dog walk way areas within Manawatu region commonly used for dog walking were sampled: The Hokowhitu, The Esplanade, Coronation Park, Milverton Park, Bledisloe Park, Albert Street, Vogel Street, Railway Road, Fitzherbert Bridge and The Bridle Track and faecal samples were also collected from the dogs that visited the Massey University Small Animal Veterinary Clinic while they defecated. Cat faecal material was collected from the Massey University Small Animal Veterinary Clinic from those cats that attended the hospital for routine vaccination and deworming procedures and from cats belonging to staff and students of Massey University, one private veterinary clinic and a commercial cattery in the Manawatu region.

### Sample collection

Each sampling site for urban wild birds and pets was visited once a month to collect faecal samples. At each sampling site fresh faecal material (moist and slimy; n = 12 per site) was collected from ducks from areas where the ducks rested while for the starlings nesting areas were identified at each sampling site and faecal samples (n = 12 per site) under each nesting area were collected during early morning hours and late evening hours. As the Canadian geese were sighted only during the months of August 2008, February, March, April and July 2009, samples could not be collected for the entire study. Likewise, swans were sighted only during the month of April 2008 and Pied stilts were sighted only during May 2008 sampling round and hence samples from these two species could not be collected for the entire study (n = 2 each species). Similarly, samples could not be collected from cats for the entire study period as it was done for the ducks, dogs and starlings. A total of 906 samples from ducks, 835 from starlings and 23 from Canadian goose for urban wild birds and 498 from dogs (n = 3 per site; n = 30 per month and n = 25 from Massey University Small Animal Veterinary Clinic) and 82 (small animal clinic: n = 77; staff and students: n = 3; cattery: n = 2) from cats were collected in transport media (Amies charcoal, Fort Richards, Auckland) from the sampling sites. During few sampling rounds, it was not possible to collect a complete set of dog samples from the faecal bins as well as for ducks and starlings (Table 1).

**Table 1:** **Overall faeco-prevalence estimates of** ***Campylobacter*** **and** ***C. jejuni*** **in duck, starling, goose, dog and cat faecal material**

Species	***n***	***Campylobacter*** positive	***C. jejuni*** positive
		(%; 95% CI)	(%; 95% CI)
Overall - urban wild birds	1,768	33 (19-51)	19 (17-21)
Ducks	906 (n = 12 per site)*	29 (26-32)	20 (18-23)
Starlings	835 (n = 12 per site)*	41 (38-45)	18 (16-21)
Geese	23 (n = 5 per month)^§^	9 (11-29)	9 (11-29)
Pied stilt	2^ϒ^	50 (1-98)	0 (0-91)
Swan	2^ϒ^	50 (1-98)	0 (0-91)
Overall - pets	580	17 (14-20)	7 (5-9)
Dogs	498 (n = 3 per site)^¥^	13 (10-16)	5 (4-8)
Cats	82	9 (4-17)	7 (3-15)

n: Number of samples.

CI: Confidence interval.

n: Number of samples.

CI: Confidence interval.

*: Ducks: For sampling rounds April 2008 to July 2008, a complete set of 12 samples could not be collected from all sites and for August 2008 sampling round: it was not possible to collect 12 samples from the Hokowhitu sampling site.

^§^: Geese: August 2008 sampling round: only two samples could be collected from the Hokowhitu sampling site.

^ϒ^: During sampling rounds April 2008 and May 2008 two swans and two pied stilts were sighted at the Massey University sampling site and samples were collected from them.

^¥^: Dogs: January 2009 sampling round: No samples could be collected from the Milverton Park site and only one sample could be collected from Coronation Park for the June 2009 sampling round. A total of 25 samples were collected from small animal clinic, Massey University.

### Bacterial isolation and DNA preparation

Faecal samples from all sources were transported immediately to the Hopkirk Research Institute Laboratory on the Massey University campus at Palmerston North and were directly streaked onto modified charcoal cefoperazone - deoxycholate (mCCDA) (Fort Richards, Auckland) plates. The inoculated plates were incubated for 48 hours at 42°C in a microaerophilic chamber (MACS VA500 Microaerophilic workstation, Don Whitley Scientific) and the presumptive *Campylobacter* colonies were sub-cultured onto blood agar plates (horse lysed blood agar, Fort Richards) for 48 hours at 42°C. The pure colonies isolated from the horse blood agar plates tested for oxidase reduction (oxidase strips, Fort Richards, Auckland), indicated by a purple colouration, were transferred to 1 mL of 2% (weight/volume) Chelex solution (Sigma-Aldrich) in distilled water and boiled at 100°C on heating blocks for 10 minutes. These were then cooled to room temperature, centrifuged at 13,000 rpm for 10 minutes and the supernatants were collected in fresh sterile eppendorf tubes and stored at -20°C.

### Polymerase chain reaction (PCR)

Isolates were characterised for *Campylobacter* spp. and *C. jejuni* using monoplex PCR using 16s rRNA gene primers for *Campylobacter* spp. and the membrane associated protein A (mapA) gene primers for *C. jejuni*, [13, 23] respectively. *Campylobacter* genus primer sequences were: forward 5′ GGATGACACTTTTCGGAGC 3′; reverse 3′ CATTGTAGCACGTGTGTC 3′. *C. jejuni* primer sequences were: forward 5′ CTTGGCTTGAAATTTGCTTG 3′ and reverse 3′ GCTTGGTGCGGATTGTAAA 5′. The PCR conditions consisted 96°C for 2 minutes for initial denaturation, 96°C for 30 seconds, primer annealing at 56°C for 30 seconds, and extension at 72°C for 30 seconds, with a final extension for 4 minutes for 35 cycles. The PCR reaction mix (Invitrogen) was prepared with 2 μL 10x PCR buffer (final concentration 1x); 2 μL dNTPs (final concentration 2 mM); magnesium chloride 1 μL, (final concentration 2.5 mM); primers 2 μL each (final concentration 1 mM); Taq DNA polymerase 0.2 μL (final concentration 1 unit per reaction); DNA 2 μL (final concentration 10 ng per μL). The reaction mix was made up to 20 μL with distilled water. The amplicons were examined by agarose gel electrophoresis with results captured using a Bio-Rad gel documentation system (Life Science Group, Canada). The isolates were tested only for *C. jejuni* as the main aim of the project was to characterise *C. jejuni* from urban wild birds and pets and hence other non-jejuni Campylobacters were not characterised.

## Results

### Faeco-prevalence of *Campylobacter*spp. and *C. jejuni*

The overall faeco-prevalence of *Campylobacter* spp. in the sampled urban wild birds was 33% (95% confidence interval [CI] 19 to 51%) and of *C. jejuni* was 19% (CI 17 to 21%). The overall *Campylobacter* and *C. jejuni* prevalence for pets were 17% (CI 14 to 20%) and 7% (CI 5 to 9%), respectively (Table 1).

### Faeco-prevalence over time

**Figure 1:**
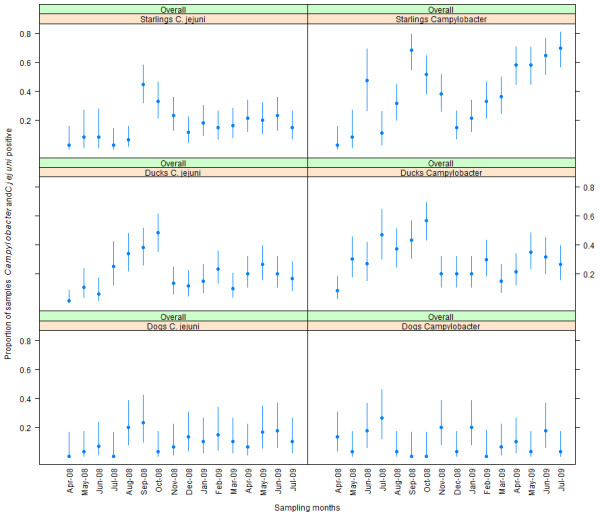
**Faeco-prevalence of** ***Campylobacter*** **and** ***C. jejuni*** **in dogs, ducks and starlings over time.** The faeco-prevalence was estimated for different months of sampling for dogs, ducks and starlings by aggregating the sampling sites.

Overtime the faeco-prevalence of *Campylobacter* spp. and *C. jejuni* differed significantly between dogs, ducks and starlings. The data from goose, swans, pied stilts and cats could not be compared with other species due to small sample sizes as well as unavailability of samples for the entire study. Hence an overall faeco-prevalence was estimated for these species and presented in Table 1. The general trend was that the faeco-prevalences of both *Campylobacter* and *C. jejuni* were relatively high during warmer months (September, October, November, and January) over the cooler months. However, there was an increase in the *Campylobacter* faeco-prevalence in starlings during the cooler months (April, May, June and July), Figure 1.

### Faeco-prevalence at different sampling sites

The Canadian goose were sighted only in the Hokowhitu sampling site where other sites had only ducks and therefore the faeco-prevalence of *Campylobacter* and *C. jejuni* reported in the study for goose represents only the Hokowhitu sampling site. Likewise swans and pied stilts were sighted only at Massey University sampling site, hence the prevalence represents only Massey University sampling site. It is difficult to categorise the sampling sites for cats as the majority of the samples were collected from the small animal veterinary clinic. The prevalence estimates for different sampling sites for ducks and starlings and dogs are presented in Tables 2 and 3, respectively. The *Campylobacter* spp. as well as *C. jejuni* faeco-prevalence in sites including The Esplanade, Memorial park and The Square were found to be relatively high compared with The Hokowhitu and Massey University sites, however, the starlings from The Hokowhitu site showed relatively high prevalence over ducks from the same site. The faeco-prevalence of *Campylobacter* spp. in dogs in The Esplanade, Bledisloe Park, Fitzherbert Bridge and the Milverton Park were relatively higher than that of other sites while the dog samples from the Bledisloe Park and The Bridle Track showed high *C. jejuni* faeco-prevalence compared to other sites.

**Table 2:** **Faeco-prevalence estimates of** ***Campylobacter*** **and** ***C. jejuni*** **in ducks and starlings at different sampling sites**

Sampling sites	Ducks ***Campylobacter*** positive	Ducks ***C. Jejuni*** positive	Starlings ***Campylobacter*** positive	Starlings ***C. Jejuni*** positive
	(%; 95% CI)	(%; 95% CI)	(%; 95% CI)	(%; 95% CI)
The Esplanade	38 (31 - 45)	25 (19 - 32)	48 (40 - 56)	25 (18 - 32)
The Hokowhitu	20 (15 - 27)	17 (12 - 23)	39 (31 - 47)	13 (8 - 19)
Memorial park	32 (26 - 40)	22 (16 - 29)	48 (40 - 56)	22 (16 - 30)
Massey University	22 (16 - 29)	15 (10 - 21)	36 (29 - 44)	13 (8 - 19)
The Square	34 (27 - 42)	24 (18 - 31)	37 (30 - 45)	20 (14 - 27)

CI: Confidence interval.

**Table 3:** **Faeco-prevalence estimates of** ***Campylobacter*** **and** ***C. jejuni*** **in dogs at different sampling sites**

Sampling sites	Dogs ***Campylobacter*** positive (%; 95% CI)	Dogs ***C. jejuni*** positive (%; 95% CI)
Albert Street	8 (2-18)	6 (1-16)
Bledisloe Park	18 (9-31)	10 (3-21)
The Bridle Track	10 (3-21)	8 (2-18)
Coronation Park	6 (1-16)	4 (0.4-13)
The Esplanade	25 (14-40)	4 (0.4-13)
Fitzherbert Bridge	16 (7-29)	6 (1-16)
The Hokowhitu	6 (1-16)	6 (1-16)
Milverton Park	12 (0.4-24)	4 (0.4-13)
Railway Road	4 (0.4-13)	4 (0.4-13)
Vogel Street	10 (3-21)	2 (0.1-10)

CI = Confidence interval.

## Discussion

Generally birds are referred to as natural reservoirs of *Campylobacter* spp. [2] and wild birds are known vectors of transmitting *Campylobacter* spp. to poultry, cattle and humans [24–26]. Similarly, pet ownership (particularly dogs and cats) has been identified as a risk factor for human campylobacteriosis particularly among small children and infants [27, 28]. The aim of this study was to estimate the faeco-prevalence of *Campylobacter* spp. and *C. jejuni* in urban wild birds, dogs and cats at different sites and over different months of sampling.

The overall faeco-prevalence of *Campylobacter* spp. was estimated to be 33% (95% CI 19 - 51%) for wild birds. The *Campylobacter* faeco-prevalence in urban wild birds is relatively high compared to migrating birds (22%) and relatively low compared to aquatic birds that feed on invertebrates (50%) [25]. However, the estimated faeco-prevalence of *Campylobacter* spp. (41%) in European starlings was relatively high compared to previous studies that ranged from 33% to 40% [16, 24, 25, 29], where the prevalence of *Campylobacter* spp. in starlings is noted to be high in general [16, 29]. In contrast, the faeco-prevalence estimates of *C. jejuni* in ducks (20%) and starlings (18%) were relatively low compared with previous studies (ducks, 30.6%; starlings (29.9%) [16, 25, 29]. The differences in the prevalence estimates could be attributed to several factors including sample size, sampling techniques, type of samples tested, age of fecal material and sensitivity of the culture techniques [30, 31].

The overall faeco-prevalence of *Campylobacter* spp. in dogs was 13% (95% CI 10% to 16%) which is relatively low compared with previous prevalence studies that reported prevalence estimates ranging from 18% to 72% [32–40] and 9% of samples from cats were positive for *Campylobacter* spp.. The overall *C. jejuni* faeco-prevalence was 5% (95% CI 4% to 8%) in dogs and 7% (95% CI 3% to 15%) in cats, while the prevalence of *C. jejuni* has been reported to be 3% to 40% [41–45] in dogs and 76% in cats [40]. In the present study faecal samples were collected from dog faecal bins while samples have been generally collected from the rectum using swabs in the majority of the studies which may have resulted in low prevalence estimates as *Campylobacter* is micro-aerophilic. When *Campylobacter* spp. are exposed to adverse conditions the organisms enter a state of existence but non-culturable forms which directly impacts the recovery rate of *Campylobacter* spp. from the faecal and/or environmental samples in culture which in turn could have also influenced the recovery rate of *Campylobacter* in this study [46, 47]. Furthermore, the survival of *Campylobacter* in dog faecal material is dependent on the prevailing environmental temperature and therefore this study may under-represent the true faeco-prevalence of *Campylobacter* in dogs. It should be acknowledged that the faeco-prevalence in cats was based on a small sample of animals attending veterinary hospitals and therefore cannot be compared with the study of dog faeces and also these cats cannot be considered as the representative of the general cat population in NZ. However, as there are only few studies of *Campylobacter* spp. in cats, and none in New Zealand, the results have been reported here.

There was marked differences observed in the faeco-prevalence of *Campylobacter* spp. as well as *C. jejuni* in dogs, ducks and starlings during different months of sampling. The prevalence estimates over time with the early spring peak in ducks was in agreement with one study [48] that was carried out in goose while few other studies [25, 49] also identified shedding of *Campylobacter* spp. in the autumn at a greater level. The relatively increased faeco-prevalence during warmer months may be speculated to have an association with increased incidence rates of human campylobacteriosis in NZ during spring and summer. While increased autumn and winter shedding of *Campylobacter* cannot be ignored as there are reports of autumn-winter outbreaks [50, 51] that have been related with organic meat consumption, handling of pets with diarrhoea and farm visits during winter. It may be hypothesised that increased environmental contamination with wild birds’ faecal material and contamination from the uncleansed dog faecal bins and/or dog faeces may be additional contributors to the winter outbreaks.

Although this study has estimated the faeco-prevalence of *Campylobacter* spp. in a country such as New Zealand that has the highest campylobacteriosis notification rates, it should be acknowledged that this study has a limitation that the study was conducted only for sixteen months. Furthermore, molecular typing of *C. jejuni* isolates from all species will aid in determining urban wild birds and pets as potential sources of human campylobacteriosis. Nonetheless, this study has provided some insights into the faeco-prevalence and the temporal trend of *Campylobacter* spp. and *C. jejuni* in urban wild birds and dogs which could be correlated with the seasonality of human campylobacteriosis in NZ context, particularly in small children that use these sampling sites for play.

## Conclusion

This study estimated the faeco-prevalence of *Campylobacter* spp. and *C. jejuni* in urban wild birds (ducks, goose and starlings) and pets (dogs and cats) in the Manawatu region of lower north island of New Zealand. The faeco-prevalence varied among months of sampling and at sampling sites where the warmer months showed increased prevalence with increased winter faeco-prevalence in starlings. This study has provided insights into the possibility of wild birds and pets being potential sources of *Campylobacter* to humans and other animal species and; the environmental faecal contamination being an important public health risk, particularly to the small children that use the sampling sites for play. However, typing Campylobacters further to different species level and genotyping the isolates by employing internationally recognised genotyping techniques such as multilocus sequence typing and antigenic (*fla*A-SVR and *por*A) typing at least for *C. jejuni* from urban wild birds and pets will provide valuable insights into (1) the role of these bird and animal species as vectors in the transmission of *C. jejuni* to humans and (2) the magnitude of their contribution towards human campylobacteriosis in NZ.

### Ethics statement

This work did not involve animals.
